# Cellulose Nanofibrils and Tubular Halloysite as Enhanced Strength Gelation Agents

**DOI:** 10.3390/polym11050919

**Published:** 2019-05-24

**Authors:** Vladimir Vinokurov, Andrei Novikov, Valentina Rodnova, Boris Anikushin, Mikhail Kotelev, Evgenii Ivanov, Yuri Lvov

**Affiliations:** 1 Functional Aluminosilicate Nanomaterials Lab, Gubkin University, 119991 Moscow, Russia; vinok_ac@mail.ru (V.V.); valentinadinges@rambler.ru (V.R.); anikushin.b@gmail.com (B.A.); kain@inbox.ru (M.K.); ivanov166@list.ru (E.I.); 2 NPK Spetsburmaterialy, Zhukovskiy, 140131 Moscow, Russia; 3 Institute for Micromanufacturing, Louisiana Tech University, Ruston, LA 71272, USA

**Keywords:** gelation kinetics, sol–gel transition, water shutoff, silica sol, cellulose nanofibrils, halloysite nanotubes

## Abstract

Silica gels are widely employed in water shutoff services, making them an essential tool in oil well management. Silica nanoparticles may serve as a strengthening additive for polymer hydrogels. In this study, we look at this statement from a different angle: What additives could be used to increase the strength of silica gels? Colloidal silica gels were prepared with various additives, and gel strength was measured by a Veiler–Rebinder apparatus. We found that cellulose nanofibrils considerably increase the gel strength (from 20–25 to 35–40 kPa), which is comparable with the industrial anionic polymer Praestol 2540. Cellulose nanofibrils can be produced from cheap industrial-grade cellulose with low-cost industrial chemicals and could be partially replaced by the even less expensive halloysite nanoclay. Cellulose nanofibrils produced from renewable sources and naturally occurring halloysite nanoclay could be used as complementary reinforcing agents.

## 1. Introduction

Silica gel formation from silica nanoparticles is widely employed in water shutoff services in oil wells [1,2] and was recently proposed to be used in geothermal applications [3].

Silica nanoparticles have been employed as filler in polyacrylamide-based compositions in enhanced oil recovery [4,5,6]. A converse proposition arises naturally: What can be used to improve the properties of concentrated silica gels formed from silica nanoparticles?

Cellulose nanofibrils (CNFs) are promising materials actively studied and available as water-dispersible powders, films, or hydrogels [7]. They can be produced from a variety of renewable plant sources, including kraft bleached eucalyptus pulp [8], corn stalks [9], and algae [10]. Research into the application of CNFs as reinforcing additives has covered such areas as the paper and cardboard industry, production of polymer nanocomposites [11], development of composites with tunable mechanical properties [12], and preparation of thermo- and pH-sensitive hydrogels [13].

Halloysite nanotubes are widely employed as reinforcing agents in plastic composites [14,15,16,17], catalyst carriers [18,19], and biomedical tissue scaffolds [20,21,22]. The most exciting feature of the halloysite is its double-charged surface, which enables various strategies for the surface modification and loading of halloysite nanotubes for compatibility with hydrophilic or hydrophobic and positively and negatively charged matrices [23,24] and for the encapsulation of functional agents for corrosion protection [25,26], drug delivery [27,28], and other applications.

The formation of composite materials from cellulose nanofibrils and halloysite nanoclay was recently reported [29], highlighting the possibilities of their co-use as reinforcing agents in composite materials and gels.

The present study was aimed at the possible usage of cellulose nanofibrils and halloysite nanotubes as reinforcing additives for silica sol-based gelation compositions. We found that cellulose nanofibrils provide gel strengthening comparable with that of the synthetic polymer Praestol 2540, and could be partially substituted with halloysite, which would still provide high gel strength.

## 2. Materials and Methods

### 2.1. Materials

Silica sol Polygel ASM K3 was purchased from JSC Polycell (Vladimir, Russia). Praestol 2540 poly(acrylamide) polymer (Ashland Deutschland GmbH, Krefeld, Germany), sodium citrate dihydrate, sulfuric acid, and hydrogen peroxide were purchased from a local dealer Chimmed (Moscow, Russia). Industrial-grade cellulose was kindly provided by Solikamskbumprom (Solikamsk, Perm Krai, Russia). Halloysite nanoclay was purchased from Sigma–Aldrich (product number 685445, St. Louis, MO, USA).

### 2.2. Preparation of Cellulose Nanofibrils

Cellulose nanofibrils were prepared from the industrial-grade cellulose in the following manner: Industrial-grade cellulose was dispersed in distilled water with homogenizer Bosch MSM66110 (Bosch, China), vacuum-filtered, and placed into a tightly closed desiccator overnight to ensure even water content. The cellulose pulp was then sampled for water content determination and subjected to acidic-oxidative treatment as described in [30], with slight modifications. Briefly, concentrated sulfuric acid was added to the 2% cellulose pulp dispersion in filtered tap water (1.6 kg dry weight of cellulose) to 8 mol/kg molality and was held at 55 °C and 90 rpm for 4 h in a stirred tank reactor. The treated pulp was then filtered, washed with distilled water until the filtrate pH was higher than 6.0, and was then redispersed in water to 1.5 mass% by ultrasound treatment with a flow-through sonifier UIP1000hd (Hielscher Ultrasonics Gmbh, Berlin, Germany) and was then wet milled with a Supermasscolloider MKCA6-5J (Masuko Sangyo Co., Kawaguchi, Japan). The prepared cellulose nanofibrils dispersion was then centrifuged and dried to 20 mass% cellulose content. Preparative yield of cellulose nanofibrils was 88.7%.

### 2.3. Characterization of the Employed Materials

For the ash content determination, dried cellulose nanofibril samples in porcelain crucibles were calcined in a Nabertherm L 15/13 furnace at 525 °C according to the TAPPI (Technical Association of the Pulp and Paper Industry) method 211-om 93 [31].

Electron micrographs were acquired with a JEM-2100 transmission electron microscope (Jeol, Japan) and a JIB-4501 scanning electron microscope (Jeol, Japan). The micrographs were analyzed with an ImageJ program suite (not less than 700 particles were measured for each sample). Samples for TEM were prepared by drop-casting the appropriately diluted dispersions onto formvar grids (Ted Pella, Redding, CA, USA). For SEM imaging, the specimens were either drop-cast (as dispersions) onto the aluminum stub or frozen with liquid nitrogen (as solidified gels), broken into pieces, and mounted onto the aluminum stub with carbon tape. All SEM specimens were then sputtered with gold (approx. 5-nm thickness) to ensure their good surface conductivity, using a JFC-1600 magnetron sputtering device (Jeol, Tokyo, Japan).

Zeta potential was measured with an SZ-100 particle size analyzer (Horiba, Kyoto, Japan) by the electrophoretic light scattering method.

### 2.4. Preparation of Reinforced Silica Gels

Reinforced silica gels were prepared using Polygel ASM K3 industrial-grade silica sol, which has a density of 1.180–1.220 g/cm^3^, pH of 8.5–9.5, SiO_2_ content of 25.0–32.0 mass%, particle size of 9–11 nm, BET (Brunauer-Emmett-Teller) surface area of 250–300 m^2^/g, and viscosity of 10 mPa·s.

Silica sols were mixed with the additives (anionic polymers, halloysite, and cellulose nanofibrils) and the gelation activator (2.4 mass% sodium citrate) using mechanical stirring and ultrasound treatment in a VBS-3DS ultrasonic bath (Vilitek, Moscow, Russia) for 30 min. Portions of the gelation mixture were then placed in vessels in preparation for the gel strength measurements.

### 2.5. Gel Strength Measurements

**Figure A1:**
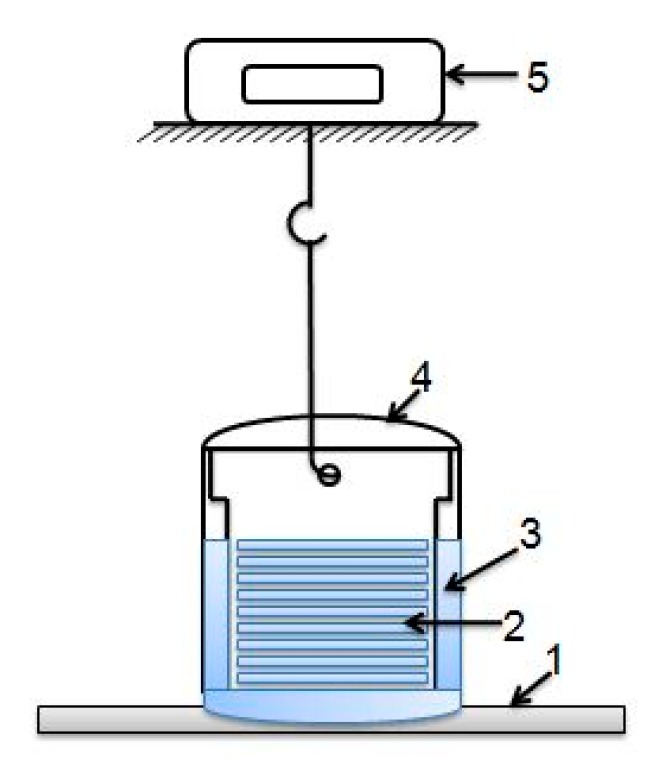
Scheme of the Veiler–Rebinder apparatus for the measurements of gel strength. 1, Z-stage; 2, perforated carbon steel plate; 3, gel; 4, vessel; 5, analytical balance.

After the gel portions had solidified in the vessels, with the special perforated plate immersed, gel strength measurements were performed at certain time intervals. The scheme of the employed apparatus is shown in Appendix A (Figure A1). Gel strength was measured by the Veiler–Rebinder method based on the extraction of the patterned or perforated plate from the gel [32], which is more suitable for the modeling of shear stress of gels in oil well conditions than the routinely employed method of inserting a probe into the gel [33]. Time intervals were counted from the moment of the addition of the gelating activator (sodium citrate).

## 3. Results

### 3.1. Characterization of Cellulose Nanofibrils

The prepared cellulose nanofibrils dispersion was a viscous white liquid with profound thixotropic properties. In several days, the dispersion formed a thick gel. After concentrating up to 20% cellulose dry weight, the cellulose nanofibrils became a wet white solid, resembling powdered cellulose. This solid mass could be redispersed with sonication into the initial gel-forming liquid.

**Figure 1:**
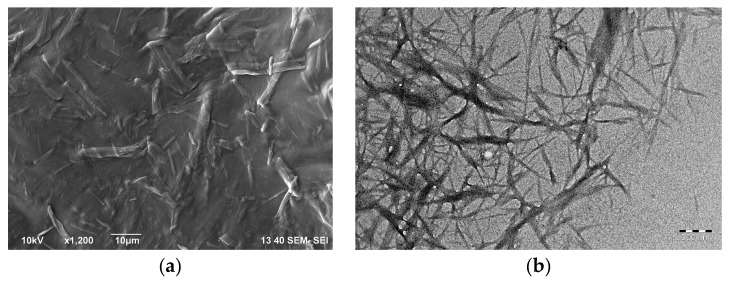
(**a**) SEM micrograph of cellulose nanofibrils. Scale bar, 10 µm; (**b**) TEM micrograph of cellulose nanofibrils. Scale bar, 200 nm.

Electron micrographs of the prepared cellulose nanofibrils are shown in Figure 1. As one can see in Figure 1, cellulose nanofibrils tend to agglomerate into fibers up to 10 µm in diameter when dried at the substrate.

**Figure 2:**
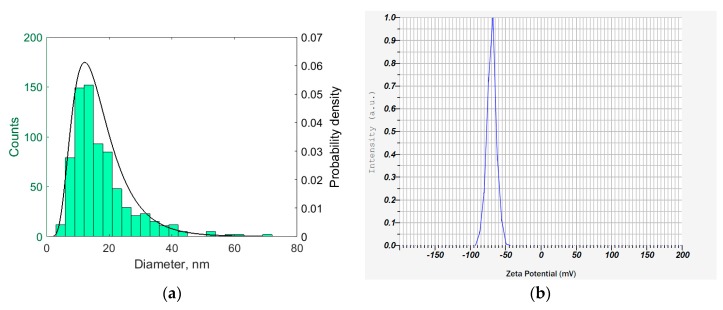
(**a**) Diameter distribution of cellulose nanofibrils; (**b**) Zeta potential distribution of cellulose nanofibrils.

Analysis of the TEM micrographs of the cellulose nanofibrils showed that the diameter of cellulose nanofibrils was well described by lognormal distribution with the parameters µ = 2.7287 and σ = 0.4778. The histogram and the fitted distribution are shown in Figure 2a. The length of the cellulose nanofibrils was hard to assess, because nanofibrils intertwine easily, even when deposited in a loose layer from the diluted dispersion, as shown in Figure 1b. While the smallest cellulose nanofibrils were around 200 nm in length, the bigger nanofibrils were longer than 1 µm.

The electrophoretic mobility of the cellulose nanofibrils was −5.6 × 10^−8^ m^2^V^−1^s^−1^, which corresponded to the apparent mean zeta potential of −69.7 mV. The reconstructed zeta potential distribution is shown in Figure 2b. Despite the inherent inaccuracy of the zeta potential reconstruction by the Smoluchowski equation (in the approximation of spherical particles), the estimated zeta potential value reflected the considerable negative charge of the cellulose nanofibrils. Indeed, the cellulose nanofibril dispersion was rather stable and showed only minor sedimentation after several weeks of storage, while gentle shaking restored the homogeneity of the dispersion.

### 3.2. Characterization of Halloysite Nanotubes

Halloysite nanotubes are a white powder, easily dispersed in water. Typically, their aqueous dispersion can be stable for up to 10 min. It is worth noting that, in this study, after some halloysite precipitated, the liquid above the sediment still contained small fractions of halloysite. In the diluted dispersion, the halloysite nanotubes had electrophoretic mobility of −3.1 × 10^−8^ m^2^V^−1^s^−1^, which corresponded to the apparent mean zeta potential of −40.0 mV.

**Figure 3:**
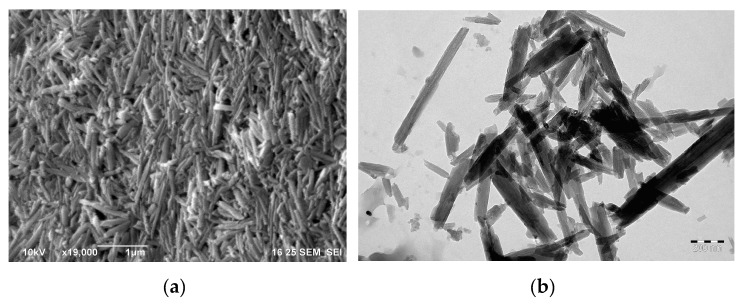
(**a**) SEM micrograph of halloysite nanotubes. Scale bar, 1 µm; (**b**) TEM micrograph of halloysite nanotubes. Scale bar, 200 nm.

Electron micrographs of the halloysite nanotubes are shown in Figure 3. As one can see in Figure 3, the halloysite nanotubes formed a dense layer when dried on a substrate, while the more dispersible fraction of halloysite aggregated in loose clusters of nanotubes, visible in the TEM micrograph (Figure 3b).

**Figure 4:**
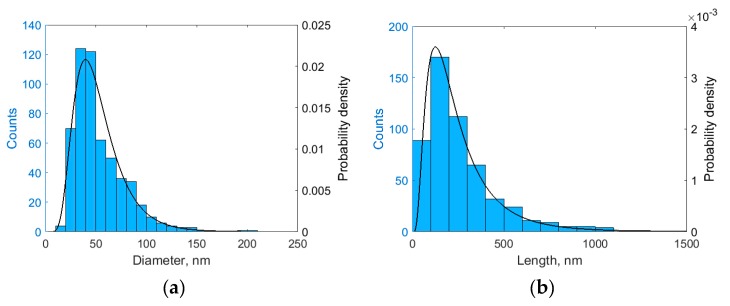
(**a**) Diameter distribution of halloysite nanotubes; (**b**) Length distribution of halloysite nanotubes.

The fraction of halloysite nanotubes visualized by TEM micrographs was characterized by length and diameter distributions. Both length and diameter were well described by lognormal distribution with the parameters µ = 3.8734 and σ = 0.4390 for diameter, and µ = 5.3164 and σ = 0.6910 for length. The histograms and the fitted distributions are shown in Figure 4.

**Figure A2:**
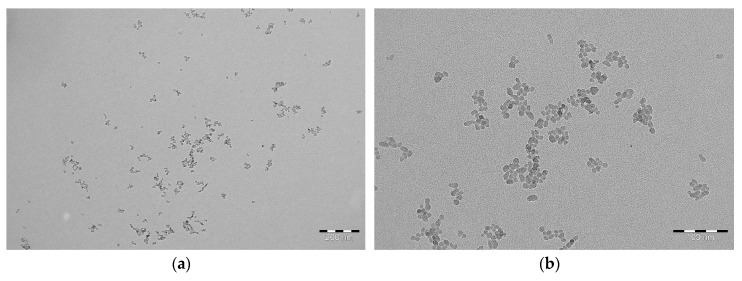
(**a**) TEM micrograph of the silica sol before gelation. Scale bar, 200 nm. (**b**) TEM micrograph of the silica sol before gelation. Scale bar, 100 nm.

Both the cellulose nanofibrils and halloysite nanotubes were much larger than the silica sol particles (7–9 nm; shown in Figure A2 in Appendix A) employed in the gelation compositions for the isolation squeeze in well servicing and could therefore be useful as reinforcement agents.

### 3.3. Gelation Kinetics and Gel Strength

**Figure 5:**
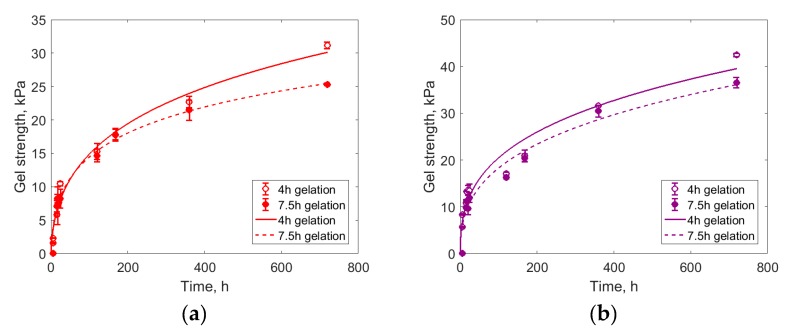
(**a**) Silica gelation without additives; (**b**) Silica gelation with 0.05% Praestol 2540 added. Gelation times were adjusted by sodium citrate dosage. Lines are Gompertz model fits.

It is known that the addition of anionic polymers to silica sol leads to the formation of reinforced gel after gelation. One example of a particularly successful reinforcement is the usage of Praestol 2540 poly(acrylamide)-based polymer. In practice, water shutoff compositions must have a low enough viscosity, so the maximum amount of polymer should not exceed 0.05 mass%. Indeed, silica gel with added 0.05 mass% of Praestol 2540 is stronger than silica gel without additives, both in the slow (7.5 h) and fast (4 h) gelation regimes, as is shown in Figure 5.

Gelation kinetics are not readily described in terms of the simplest models (such as pseudo-first-order or pseudo-second-order kinetics) but can be approximated with a Gompertz-like model:

(1) $$s=a_{1}\cdot e^{-a_{2}e^{-a_{3}\log t}}$$

where *s* is gel strength, *t* is time, and *a*_1_–*a*_3_ are fitted parameters. The model reflects the rapid onset of the gel strength after the gelation time and the slow growth of the gel strength during its ripening. In Figure 5, the lines are Equation (1) fits that approximate the experimental points. The fitted parameters for Equation (1) are listed in Table A1 in Appendix A.

**Figure 6:**
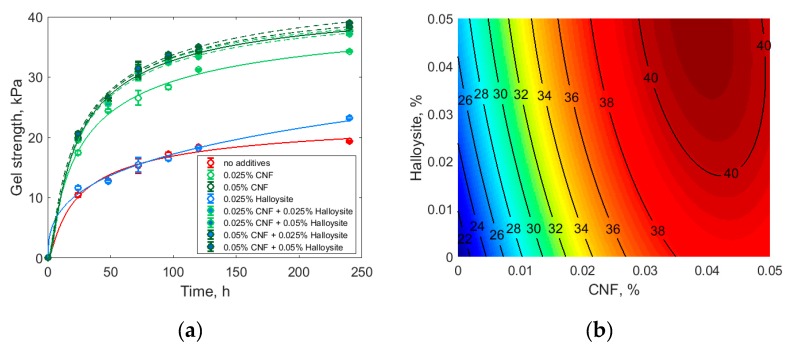
(**a**) Silica gelation with various additives. Lines are Gompertz model fits. (**b**) Gel strength at 240 h (showed by isolines, kPa) as a function of cellulose nanofibrils and halloysite added. CNF: cellulose nanofibrils.

The addition of cellulose nanofibrils and halloysite increased the gel strength, as shown in Figure 6a. One would expect a more profound strengthening effect from the halloysite nanotubes at 0.05 mass% loading, because this value corresponded to the volume fraction of halloysite nanotubes of 0.024 vol.% or approximately 1.5 nanotubes/µm^3^ (assuming the ideal dispersibility of halloysite, density of halloysite of 2.53 g/cm^3^, density of silica sol of 1.20 g/cm^3^, halloysite length of 126.3 nm, and halloysite diameter of 39.7 nm). Apparently, the halloysite was not uniformly distributed throughout the silica sol even after prolonged sonication.

Interestingly, the simultaneous addition of cellulose nanofibrils and halloysite increased the gel strength more than their separate addition, possibly reflecting the difference in the characteristic size of these additives. The influence of both additives was approximated as the quadratic model:

*s* = *a*_1_*X* + *a*_2_*X*^2^ + *a*_3_*XY* + *a*_4_*Y* + *a*_5_*Y*^2^ (2)

where *s* is gel strength at 240 h after gelation, *X* is cellulose nanofibrils content (in mass%), *Y* is halloysite content (in mass%), and *a*_1_–*a*_5_ are fitted parameters. As a result of fitting, the following model was obtained:

*s* = 19.426 + 833.30*X* + 9452.6*X*^2^ − 1985.1*XY* +173.58*Y* − 976.57*Y*^2^, (3)

where the designations are as in Equation (2). The significance of the fitted parameters was checked by Student’s statistics. For every parameter, the probabilities derived from the Student’s statistics were less than 0.05, so the model did not need refinement. The fitted model is shown in Figure 6b.

**Figure A3:**
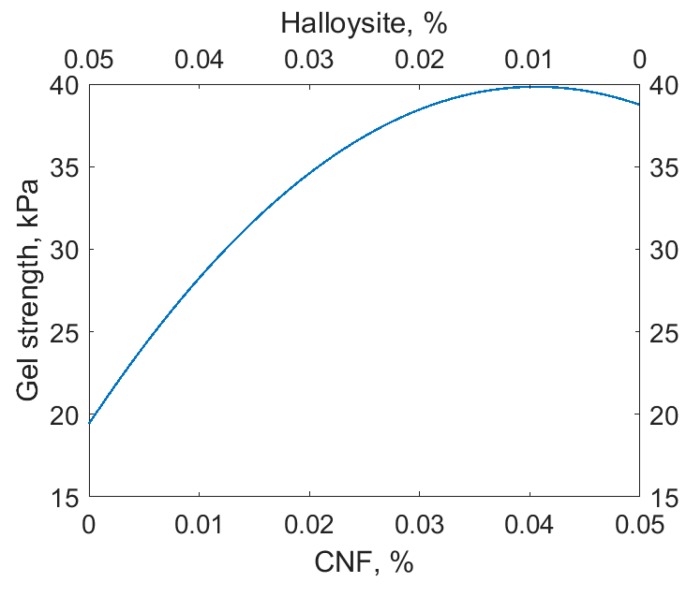
Modeled gel strength at 240 h for the total added amount of CNF and halloysite of 0.05 mass%.

The addition of CNF and halloysite in various combinations increased the gel strength almost to the same level, around 35–40 kPa. Considering the practical limit of 0.05 mass% of the total additives, we can conclude that about 0.02 mass% of CNF may be replaced with much cheaper halloysite with the same gel strength (see also Figure A3 in Appendix A). In this regard, the best gelation mixture composition we identified was 0.03 mass% CNF and 0.02 mass% halloysite in the silica sol.

**Figure 7:**
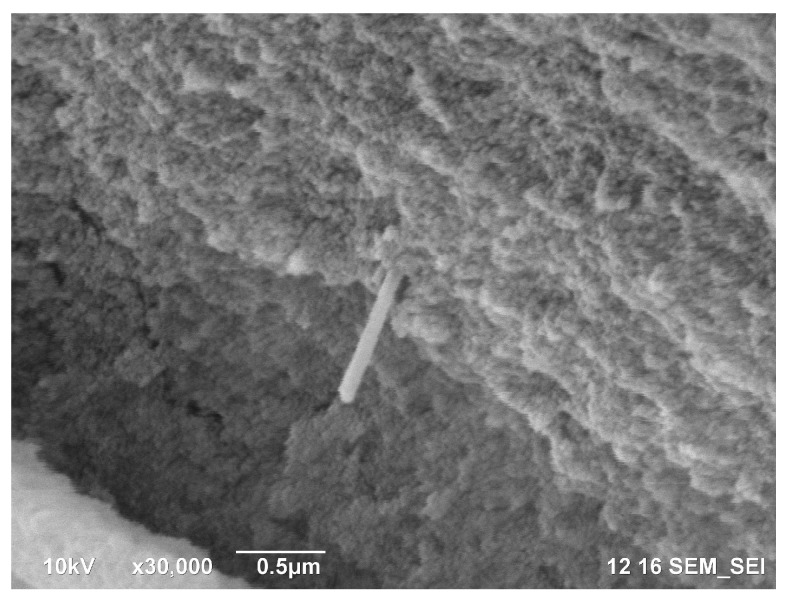
SEM micrograph of the silica gel with 0.05% halloysite cleaved in liquid nitrogen. The halloysite nanotube is visible in the center of the image. Scale bar, 500 nm.

**Figure A4:**
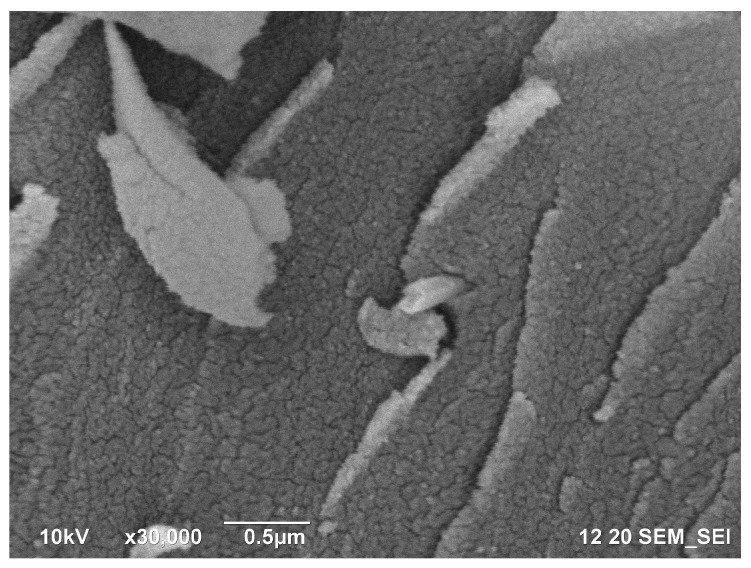
SEM micrograph of the silica gel with 0.05% halloysite cleaved in liquid nitrogen. The halloysite nanotube is visible in the center of the image. Scale bar, 500 nm.

The reinforcement of the silica gel by the halloysite nanotubes is confirmed by the SEM images of the rapidly frozen and cleaved gel (see Figure 7 and Figure A4 in Appendix A).

## 4. Discussion

In agreement with previous studies, sodium citrate induces the sol-gel transition in silica sols with the gelation time being controlled by the sodium citrate concentration [34]. Here, the most durable gels formed when the gelation time was rather short (4 h), but a further decrease in gelation time was not practical, considering the time necessary for the gelation mixture application in the oil well. Both with slow and short gelation, reinforced gels demonstrated higher strength; this increase in strength was detectable at short gel ripening times (around 240 h).

The cellulose nanofibrils produced from the industrial-grade cellulose reinforced the silica gel almost as well as the synthetic polymer Praestol 2540. The halloysite nanotubes also increased the gel strength but not as much as CNF or Praestol 2540. Nevertheless, the combined reinforcement action of CNF and halloysite was comparable to that of the synthetic polymer Praestol 2540. At high CNF loading, halloysite additionally increased the gel strength, thus making possible at least partial replacement of CNF in the reinforcement additive composition.

The reinforcing effect of the studied additives may depend on the characteristic particle size; while Praestol 2540 molecules, which are comparable in size with fine silica sol particles and are known for the efficient removal of fine colloidal particles from water [35], CNF, and especially halloysite, are considerably larger. One would expect high reinforcing ability from the larger fillers, but this effect has been observed only in matrices that are rigid enough, such as in block polymers [36]. Smaller CNF have a higher affinity for the silica particle surface and thus demonstrate a higher reinforcing effect. The different scale of their action may explain the observed synergism of CNF and halloysite in silica gel strengthening. While large halloysite nanotubes provide the rigidity of the gel in the micrometer-size range, CNF bind the silica sol particles, forming intertwined elastic threads in the submicrometer range.

The complementary scales of CNF and halloysite action could be further investigated by the study of the synergism of gel strengthening additives of different sizes, e.g., the study of the combined anionic polymer/CNF/halloysite additive. The observed synergism in the case of a triple additive would confirm the hypothesis of the complementarity of differently sized additives.

Another possible explanation for the different strengthening abilities of the studied additives is their different zeta potential. Weakly charged halloysite nanotubes provide small reinforcing effect, whereas strongly charged CNF and anionic polymers increase the gel strength considerably. The gelation process could involve the formation of joint silica–additive bridge bonds with counter-ions (typically, alkali metal ions). Strongly charged additives could form these bridge bonds more efficiently, thus providing higher gel strength. To test this hypothesis, the study of the reinforcing ability of differently charged halloysite nanotubes is highly desirable. If the gel strengths were found to be dependent on the zeta potential of the halloysite nanotubes, it would confirm this hypothesis.

The observed reinforcing effect of CNF and halloysite could be employed not only in the gelation compositions for oil well services but also in other industrial gelation products. Additional investigations could open up possibilities for the reinforcement of various materials by additives being hierarchically organized by size.

The economic effect of the observed results is rather promising, because, despite the relatively high cost of commercially available CNF, we can produce it from cheap industrial-grade cellulose with low-cost industrial chemicals (sulfuric acid and hydrogen peroxide). Furthermore, CNF could be partially replaced by even cheaper halloysite—a natural product available in thousands of tons at a reasonable price.

## Appendix A

**Table A1:** The fitted parameters for Equation (1).

Experimental conditions	a_1_	a_2_	a_3_
**Figure 5**			
No additives; gelation time 4 h	412.89 ^1^	5.7940	0.12076
No additives; gelation time 7.5 h	101.80	4.3339	0.17328
0.05% Praestol 2540; gelation time 4 h	19395	8.6720	0.051108
0.05% Praestol 2540; gelation time 7.5 h	44238	9.6360	0.046180
**Figure 6**			
No additives	24.940	6.0196	0.59552
0.025% CNF^2^	43.437	5.7342	0.58211
0.05% CNF	44.446	7.6086	0.69905
0.025% Halloysite	4322.1	7.2512	0.059066
0.025% CNF + 0.025% Halloysite	44.246	7.2674	0.68397
0.025% CNF + 0.05% Halloysite	43.803	8.6246	0.74702
0.05% CNF + 0.025% Halloysite	45.458	7.4676	0.69053
0.05% CNF + 0.05% Halloysite	48.352	6.0964	0.61393

^1^ Note that in contrast to the original Gompertz model, the parameter a_1_ in Equation (1) is not necessarily the asymptotic value at *t*→∞. ^2^ CNF—cellulose nanofibrils
